# Engineered *Salmonella* Enteritidis vector targeting innate immune molecules provides protection against *Salmonella* Enteritidis and *Salmonella* Typhimurium

**DOI:** 10.1016/j.psj.2025.105724

**Published:** 2025-08-24

**Authors:** Wenjing Li, Yu-an Li, Yuqin Zhang, Huoying Shi

**Affiliations:** a College of Veterinary Medicine, Yangzhou University, Yangzhou 225009, Jiangsu, China; b Jiangsu Co-Innovation Center for the Prevention and Control of Important Animal Infectious Diseases and Zoonoses, Yangzhou, China; c Joint International Research Laboratory of Agriculture and Agri-Product Safety, Yangzhou University (JIRLAAPS), Yangzhou, China

**Keywords:** Live attenuated *Salmonella* vaccine, *Salmonella* mRNA interferase-regulating vector, cGAS–STING innate immune pathway, vaccines development

## Abstract

Targeting the innate immune response is a critical factor in vaccine success. There are numerous serotypes of *Salmonella*, and cross-reactivity among different serotypes or even strains within the same serotype is limited. This makes it difficult for vaccines to provide broad protection, posing a challenge to effective prevention and control. In this study, a *Salmonella* Enteritidis (*S.* Enteritidis) candidate vaccine strain, rSC0163, with high immunogenicity, was constructed by introducing the *Salmonella* mRNA interferase-regulating vector (SIRV) system. The rSC0163 strain, enhanced by the SIRV system, targets the host's innate immune cyclic GMP-AMP synthase (cGAS) molecule, triggering downstream immune pathways and significantly increasing levels of antibodies (IgA and IgY) and cytokines (IFN-γ and IL-4) in the peripheral blood of immunized chickens. Compared with the control strain rSC0162, the antiserum from chickens immunized with rSC0163 showed significantly enhanced opsonophagocytic activity. The highest opsonophagocytic assay (OPA) titers against the *S.* Enteritidis C50041 strain, *S.* Enteritidis CVCC3949 strain, and *Salmonella* Typhimurium (*S.* Typhimurium) UK-1 strain reached 1:256, 1:128, and 1:64, respectively. Furthermore, rSC0163 provided 100 % and 83 % protection against lethal doses of the C50041 and CVCC3949 strains, respectively, and 66 % protection against the heterologous *S.* Typhimurium UK-1 strain. These protection rates were significantly higher than those induced by rSC0162. In conclusion, integrating the SIRV system into the attenuated *S.* Enteritidis vector enhances both innate and adaptive immune responses, offering a basis for developing cross-serotype protective vaccines and *Salmonella*-based delivery platforms targeting other pathogens.

## Introduction

*Salmonella enterica*, a facultative intracellular pathogen, can cause disease in animal hosts (Aviv et al., 2016; Foley et al., 2008). To date, over 2600 *Salmonella enterica* serovars have been identified. Epidemiological evidence indicates that *Salmonella* Enteritidis (*S.* Enteritidis) and *Salmonella* Typhimurium (*S.* Typhimurium) are the predominant serovars associated with outbreaks of avian salmonellosis (Eeckhaut et al., 2018; Kipper et al., 2022; Petsong et al., 2019). Antibiotics are commonly used as an effective treatment for bacterial infections; however, improper use may lead to increased resistance in *Salmonella* strains (Hu et al., 2020; Martinez, 2009). In addition, early-life exposure to antibiotics has been shown to delay the microbiota maturation in broilers and disrupt inter-microbial interactions (Gao et al., 2017). Consequently, there is a pressing need to develop alternative strategies to combat avian salmonellosis and reduce reliance on antibiotics.

Vaccines, as an alternative to antibiotics, represent an effective strategy for preventing salmonellosis in the poultry industry (Lalsiamthara et al., 2016; Nandre et al., 2015). Currently, subunit and inactivated *Salmonella* vaccines provide limited cross-protection between different serotypes (Huberman et al., 2022; Kang et al., 2022; Lei et al., 2023; Zhang et al., 2022). To some extent, live attenuated *Salmonella* vaccines can address these limitations. Genetically engineered live attenuated *Salmonella* vaccines exhibit specific adjuvant properties that induce a comprehensive immune response, including humoral, mucosal, and cellular immunity (Lalsiamthara et al., 2016; Li et al., 2023a; Nandre et al., 2015). However, replication of attenuated *Salmonella* vectors in the host may lead to the release of vaccine strains into the environment via immunized animals under certain conditions (e.g., incomplete attenuation or high-dose administration), posing potential biosafety risks (Frey, 2007). Conversely, excessive attenuation may reduce immunogenicity, ultimately resulting in vaccine failure. Therefore, ensuring both safety and immunogenicity is essential for the development of effective live attenuated *Salmonella* vaccines.

Activation of innate immunity is crucial for enhancing vaccine immunogenicity, and innate immune agonists are commonly incorporated into vaccines to stimulate pattern recognition receptors (PRRs) (Khurana et al., 2011; Silva et al., 2021; Wen et al., 2023). For example, the AS01 adjuvant in the Shingrix® vaccine activates TLR4 through MPL and promotes dendritic cell maturation via QS-21, thereby facilitating the maturation of antigen-presenting cells (APCs) (Chaudhury et al., 2020; Nielsen et al., 2021). The CpG DNA adjuvant in the HEPLISAV-B**®** vaccine acts as a TLR9 agonist to activate APCs (Pillet et al., 2022; Yang et al., 2014). The cyclic GMP-AMP synthase (cGAS)-stimulator of interferon genes (STING) pathway is a recently identified innate immune signaling mechanism. It detects cytosolic DNA and triggers the production of type IFN-I, pro-inflammatory cytokines, and chemokines (Pokatayev et al., 2020; Riederer et al., 2022; Wang et al., 2020). Research indicates that activation of the STING pathway can enhance the development of adaptive immunity, making it a promising target for vaccine development (Gray et al., 2012; Sanchez Alberti et al., 2017; Wang et al., 2020).

We previously developed a *Salmonella* mRNA interferase-regulating vector (SIRV) system. This system enables *Salmonella Choleraesuis* to actively lyse and release cytoplasmic DNA, thereby activating the cGAS-STING pathway and inducing the production of innate immune factors such as IFNs and other cytokines (Li et al., 2023b). In this study, the SIRV system was used to construct an *S.* Enteritidis candidate vaccine strain, designated rSC0163, which is expected to improve safety and immunogenicity for the prevention and control of multiple *Salmonella* serotypes.

## Materials and methods

### Ethical statement

Specific-pathogen-free (SPF) embryonated eggs were purchased from Boehringer Ingelheim (Beijing, China) and hatched under axenic (germ-free) conditions. Animal experiments were conducted in accordance with the regulations of the Jiangsu Administrative Committee for Laboratory Animals (SYXK [SU] 2021-0007). All procedures strictly followed the Jiangsu Laboratory Animal Welfare and Ethics Guidelines and complied with international regulations.

### Bacterial strains, plasmids, and growth conditions

Dr. Roy Curtiss III generously provided *Escherichia coli* (*E. coli*) χ7213 and plasmid pRE112 (Kang et al., 2002; Roland et al., 1999). The *S.* Enteritidis C50041 strain (LD_50_: 8.5 × 10^9^ CFU) and *S.* Enteritidis CVCC3949 strain (LD_50_: 1.4 × 10^9^ CFU), which differ in virulence, were obtained from the China Institute of Veterinary Drug (Beijing). Bacterial strains were grown in broth or on agar plates. Luria-Bertani (LB; Oxoid) or Nutrient Broth (NB; BD Difco) media were supplemented, as required, with chloramphenicol (Cm, 25 mg/mL), 2-6-diaminopimelic acid (DAP, 50 mg/mL), l-arabinose (0.2 % wt/vol), d-mannose (0.2 % wt/vol), or nalidixic acid (20 mg/mL). Mutants were selected on LB agar containing 5 % sucrose. A comprehensive list of strains and plasmids used in this study is provided in Table 1.

**Table 1:** Bacterial strains and plasmids.

Bacterial strains and plasmids	Characteristics a	Source or reference
Bacterial strains		
χ7213	*E. coli* strains, *thi‑1 thr‑1 leuB6 fhuA21 lacY1 glnV44 asdA4 recA1 RP4 2‑Tc*::*Mu pir; Km^r^*	Kang et al. (2002)
C50041	*Salmonella* Enteritidis*,* wild-type, virulent	China Institute of Veterinary Drug
CVCC3949	*Salmonella* Enteritidis*,* wild-type, virulent	China Institute of Veterinary Drug
UK-1	*Salmonella* Typhimurium*,* wild-type, virulent	Lab stock
rSC0162 (non-SIRV strain)	Δ*relA*::*araC* P_araBAD_*lacI* TT Δ*endA*::*araC* P_araBAD_*mazE* TT Δ*manA*	This work
rSC0163 (SIRV strain)	Δ*relA*::*araC* P_araBAD_*lacI* TT Δ*endA*::*araC* P_araBAD_*mazE* TT Δ*manA* Δ*cysG*:P_lac_*mazF*	This work
Plasmids		
pRE112	*oriT oriV sacB Cm^r^*	Roland et al. (1999)
pS003: Δ*relA*::*araC* P_araBAD_*lacI* TT	Suicide vector for Δ*relA*::*araC* P_araBAD_*lacI* TT, pRE112	Li et al. (2023b)
pS0131: Δ*endA*::a*raC* P_araBAD_*mazE* TT	Suicide vector for Δ*endA*::*araC* P_araBAD_*mazE* TT, pRE112	
pS0132: Δ*cysG*:P_lac_*mazF*	Suicide vector for Δ*cysG*:P_lac_*mazF*, pRE112	
rSC3879: Δ*manA*	Suicide vector for Δ*manA*, pRE112	

a Km^r^ Kanamycin resistance. Cm^r^ Chloramphenicol resistance.

### Strains construction

The construction of *S.* Enteritidis strains was performed as described previously (Lee et al., 2007; Curtiss et al., 2009). Briefly, the non-SIRV strain rSC0162 deletion mutant was generated by standard homologous recombination using the suicide plasmids pS003, pS0131, and rSC3879. Similarly, the SIRV strain rSC0163 deletion mutant was constructed from the rSC0163 deletion mutant using the suicide plasmid pS0132. The primers listed in Table 2 were used for PCR confirmation of all mutations.

**Table 2:** The primers information.

Primers name	Sequences (5′−3′) Forward primer/reverse primer	References
Δ*relA*::*araC* P_araBAD_*lacI* TT	CGGGTACCGTGATATGGGGGCATGCAACAAT/CCCAAGCTTGAGCTCGAGGGCGTTCCGGCGCTGGTAGAA	Li et al. (2023b)
Δ*endA*::*araC* P_araBAD_*mazE* TT	GGCTCTCCCCAGTCGATATT/AAACCAATTGTCCATATTGCA	
Δ*cysG*:P_lac_*mazF*	GGCGCGCTGTGTAACTGG/TGAGTTGTAATTCCTCTGG	
Δ*manA*	GGGGGTACCTTCGGCACGGAAACATGTTCGCT/GGGGAGCTCGCCGCGCTGGTAGTTTTGATAACTTAA	
*S.* Enteritidis *stn*	GTCAGCCAGGCGTGTTGTG/TCAGATGCGGAATAGAGCGATTTG	
IL-4	TGAATGACATCCAGGGAGAG/GGCTTTGCATAAGAGCTCAG	Zhang et al. (2023)
IFN-γ	AGCTGACGGTGGACCTATTATT/GGCTTTGCGCTGGATTC	
β-actin	CAACACAGTGCTGTCTGGTGG/ATCGTACTCCTGCTTGCTGATCC	

### Characterization of regulatory elements in SIRV

As described previously, the regulatory elements within the SIRV system were characterized (Li et al., 2023b). Briefly, overnight cultures of the non-SIRV strain rSC0162 and the SIRV strain rSC0163 were grown for 12 h in NB medium supplemented with arabinose. These cultures were then transferred into fresh NB medium, with or without arabinose, for a total of 20 passages. At the 2nd, 4th, 6th, 8th, 10th, 12th, 14th, 16th, 18th, and 20th passages, bacterial suspensions were diluted and plated on LB agar to determine bacterial density. Additionally, the bacterial suspension of rSC0163 was adjusted to an optical density of 0.6 at 600 nm. Western blot analysis was performed as previously described, using anti-MazE, anti-MazF, and anti-LacI antibodies (Abcam) (Li et al., 2023b).

### Selection of spontaneous nalidixic acid-resistant mutants

To analyze the colonization of *S.* Enteritidis strains in organs, the non-SIRV strain rSC0162 and the SIRV strain rSC0163 were cultured on LB medium or plates as described previously (Tzeng et al., 2006). The media were supplemented with arabinose, and the concentration of nalidixic acid was gradually increased from 5 to 100 μg/mL. After colonies appeared on LB plates containing arabinose and nalidixic acid (100 μg/mL), they were subcultured on LB plates containing arabinose for three passages. Single colonies were then inoculated on LB plates containing arabinose and nalidixic acid (100 μg/mL) to obtain spontaneous nalidixic acid-resistant mutants.

### Colonization of SIRV in chickens

The spontaneous nalidixic acid-resistant mutants of the non-SIRV strain rSC0162 and the SIRV strain rSC0163 were cultured and pelleted by centrifugation as described previously (Li et al., 2017). Twenty-four chickens were allocated into two groups. Each group was orally inoculated with either the non-SIRV strain rSC0162 or the SIRV strain rSC0163 (1 × 10^9^ CFU/200 μL). On days 3, 7, 14, and 21 post-vaccination, three chickens from each group were sacrificed. Spleen, liver, and cecal lymph node tissues were collected, weighed, and homogenized in PBS. Bacterial densities in these tissues were measured as described previously (Li et al., 2020a, 2023c).

### BrdU assay

BrdU assay was performed as previously described (Li et al., 2023b). *Salmonella* strains were cultured in BrdU (Sigma) for DNA labeling. Subsequently, RAW264.7 macrophages were infected with BrdU-labeled *Salmonella* strains. Cells were lysed in PBS containing digitonin at 0, 1, 6, 12, and 24 h after infection. Supernatants were applied to Protein-G Dynabeads (Roche), which were then washed and conjugated with anti-BrdU antibodies (Sigma). Bound BrdU-labeled DNA was released by boilingand precipitated with isopropyl alcohol. The purified DNA served as a template for detecting *S.* Enteritidis strain genes. Quantitative real-time PCR (qRT-PCR) was performed to measure relative gene expression levels, with β-actin as the internal reference for normalization.

### Macrophage infection

RAW264.7 cell infection was carried out with slight modifications to a previously described protocol (Li et al., 2023b). RAW264.7 cells (1.0 × 10^6^ cells per well) were infected with PBS, the non-SIRV strain rSC0162, or the SIRV strain rSC0163, each at 10 multiplicity of infection (MOI). Twelve hours after infection, cells were collected and lysed in cell lysis buffer. Samples were separated by SDS–PAGE, followed by Western blot analysis using anti-cGAS (Cell Signaling Technology), anti-phospho-STING (Cell Signaling Technology), anti-phospho-TBK1 (Cell Signaling Technology), and anti-phospho-IRF3 (Cell Signaling Technology) antibodies (Li et al., 2025). Interferon-stimulatory DNA (ISD) served as a positive control, and β-actin (Abcam) was used as the internal reference.

### Immunization and challenge

The non-SIRV strain rSC0162 and the SIRV strain rSC0163 were cultured and harvested by centrifugation. Chicks were divided into three groups of 18 birds each. At 1 day of age, each group was orally administered rSC0162 (1 × 10⁹ CFU/200 μL), rSC0163 (1 × 10⁹ CFU/200 μL), or sterile PBS (200 μL). A booster immunization was administered using the same method two weeks later (at 14 days of age). On day 14 after booster immunization (i.e., at 28 days of age), each group was divided into three sub-groups (six chickens per subgroup), which were intramuscularly injected with approximately 10 × LD_50_ suspensions of *S.* Enteritidis C50041 strain (8.6 × 10^10^ CFU), *S.* Enteritidis CVCC3949 strain (2.2 × 10^10^ CFU), or *S.* Typhimurium UK-1 strain (3 × 10^9^ CFU), respectively. After challenge, birds were monitored twice daily for 14 consecutive days. Mortality and clinical signs were recorded daily. When required, chicken small intestine samples were preserved in 10 % formalin buffer. Histological scores were assigned according to a previous description (Li et al., 2023d): 0 = normal; 1 = sparse inflammatory infiltrate and relatively intact intestinal villi; 2 = mild inflammatory infiltrate, intestinal villi swelling, and crypt hyperplasia; 3 = multiple inflammatory lesions with intestinal congestion and bleeding; 4 = severe inflammatory infiltrate and intestinal villi necrosis/shedding.

### Preparation of outer membrane proteins (OMPs)

The OMPs of the *S.* Enteritidis C50041 strain (SE (C50041)-OMPs), *S.* Enteritidis CVCC3949 strain (SE (CVCC3949)-OMPs), and *S.* Typhimurium UK-1 strain (ST-OMPs) were prepared as previously described (Li et al., 2017, 2020a). Briefly, wild-type *Salmonella* strains were grown in LB medium, harvested by centrifugation, and resuspended in 20 mM Tris-HCl (pH 8.6) containing 1 % sarkosyl. Bacterial lysis was achieved by sonication using a Sonics Vibra Cell apparatus. The outer membrane fraction was then collected, and the pellet was resuspended in 4 mL of 20 mM Tris-HCl buffer (pH 8.6) and stored at −80°C.

### Enzyme‑linked immunosorbent assay (ELISA)

At 2 and 4 weeks post-inoculation, sera and gut rinse samples were collected. Antibody titers were measured by ELISA. Briefly, SE (C50041)-OMPs, SE (CVCC3949)-OMPs, and ST-OMPs were coated onto 96-well polystyrene plates. After incubation, sera samples were diluted 1:50, and gut rinse samples were diluted 1:5. Plates containing sera samples were incubated with goat anti-chicken IgY–HRP antibodies (SouthernBiotech), and plates containing gut rinse samples were incubated with IgA–HRP antibodies (Abcam).

### Lymphocyte proliferation assay

Peripheral blood mononuclear cells (PBMCs) were isolated 1 week post-vaccination using the chicken PBMC isolation kit (Solarbio). PBMCs (2 × 10^5^ cells per well) were stimulated with 20 μg/mL SE (C50041)-OMPs, SE (CVCC3949)-OMPs, ST-OMPs, or Concanavalin A (ConA, 5 μg/mL) for 48 h, followed by the addition of CCK-8 reagent (Solarbio) for further incubation. The stimulation index (SI) was calculated as follows: SI = mean OD_450_ of antigen-stimulated cells/mean OD_450_ of unstimulated cells.

### Cytokine detection

PBMCs (1 × 10^7^ cells per well) were cultured and incubated in a humidified environment for 48 h with 20 μg/mL SE (C50041)-OMPs, SE (CVCC3949)-OMPs, ST-OMPs, or ConA (5 μg/mL). After incubation, mRNA expression levels of IFN‐γ and IL-4 in PBMCs were measured by qRT-PCR (Zhang et al., 2023). Primer sequences are shown in Table 2.

### Opsonophagocytic assay (OPA)

The assay was performed as previously described (Boerth et al., 2023; Li et al., 2022). Briefly, on the 7th day after booster immunization, serum samples were collected from chickens and twofold serially diluted for the OPA. Chicken neutrophils were isolated using a chicken peripheral blood neutrophil isolation kit (Solarbio). Log-phase *S.* Enteritidis C50041, *S.* Enteritidis CVCC3949, and *S.* Typhimurium UK-1 strains were incubated with serum samples for 30 min. Chicken neutrophils (1 × 10^6^ cells) were mixed with 1 × 10^6^ CFU bacteria pre-coated with the serum and incubated for 90 min. Diluted samples were then spread on LB agar plates. The OPA titer was defined as the highest serum dilution at which 50 % of the tested bacteria were eliminated.

### Statistical analysis

All experiments were performed at least three times. Statistical analyses were conducted using GraphPad Prism software. Data were compared using the Mann–Whitney U test and are reported as mean ± SD. Survival rates after challenge with wild-type *S.* Enteritidis and *S.* Typhimurium strains were evaluated using the log-rank test. *P*-values < 0.05 were considered statistically significant.

## Results

### SIRV mediates arabinose-dependent programmed self-lysis

**Fig. 1:**
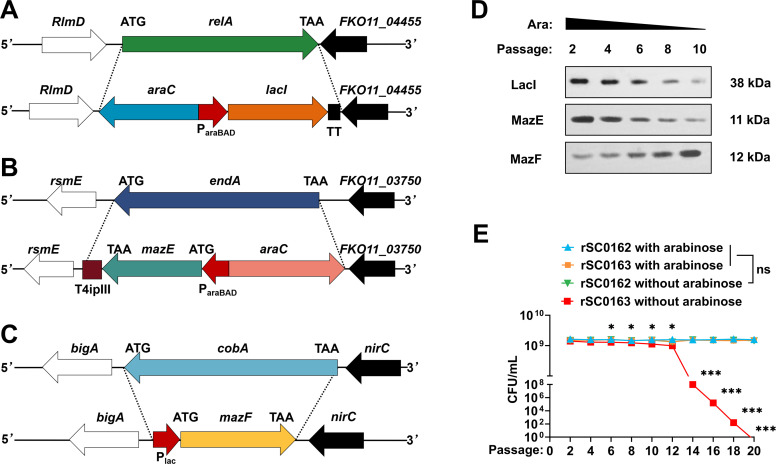
Schematic map of chromosomal mutations and phenotypic characterization of SIRV. (A) Schematic map of Δ*relA*::*araC* P_araBAD_*lacI* TT mutation. (B) Schematic map of Δ*endA*::*araC* P_araBAD_*mazE* TT mutation. (C) Schematic map of Δ*cysG*:P_lac_*mazF* mutation. (D) Western blot analysis of LacI, MazE, and MazF proteins synthesized in the SIRV system when strains are cultured in NB medium with reduced arabinose concentration. (E) Bacterial density measured in NB medium with or without arabinose. Ns, not significant. *, *P* < 0.05, **, *P* < 0.01, ***, *P* < 0.001, compared with strains rSC0162 (without arabinose) and rSC0163 (without arabinose).

The MazE antitoxin controls the expression of the MazF toxin, thereby preventing premature bacterial damage (Li et al., 2023b; Schifano et al., 2014). To achieve programmed self-lysis of the recombinant attenuated *S*. Enteritidis vector after complete colonization and stimulation of the lymphatic system, we introduced the SIRV system into the attenuated *S*. Enteritidis vector to generate SIRV strain rSC0163. The SIRV system included *relA*::*araC* P_BAD_ *lacI* TT, *endA*::*araC* P_BAD_ *mazE* TT, and *cysG*:P_lac_ *mazF* mutations (Fig. 1A-C). The synthesis of LacI, MazE, and MazF in response to arabinose in the SIRV system was evaluated by Western blot. As arabinose levels decreased in vitro, protein expression levels of LacI and MazE were downregulated, while MazF expression increased (Fig. 1D). Subsequently, we cultured the SIRV strain rSC0163 and the non-SIRV strain rSC0162 in NB medium with or without arabinose. There was no difference in bacterial density between the SIRV rSC0163 and non-SIRV strains when grown in NB medium containing arabinose. However, colony numbers of SIRV strain rSC0163 gradually decreased during serial passages in NB medium without arabinose, whereas the bacterial density of non-SIRV strain rSC0162 remained unchanged (Fig. 1E). These results indicate that in the SIRV system, the expression of LacI, MazE, and MazF is regulated by the P_araBAD_/AraC regulatory system and exhibitsarabinose-dependent delayed programmed self-lysis.

### SIRV mediates programmed self-lysis in vivo

**Fig. 2:**
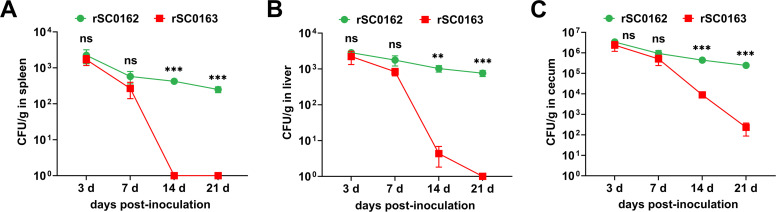
Colonization of *S.* Enteritidis strains in chickens. Chickens are orally inoculated with non-SIRV strain rSC0162 or SIRV strain rSC0163. Bacterial counts are determined from the spleen (A), liver (B), and cecum (C) tissues at 3, 7, 14, and 21 days post-infection. Colonies were recorded as CFU/g of organ samples. Data are expressed as the mean ± SD from three infected chickens. Ns, not significant, *, *P* < 0.05, **, *P* < 0.01, ***, *P* < 0.001.

Colonization represents an interaction between a live vector and lymphoid tissues and is closely associated with vector immunogenicity (Li et al., 2022). To evaluate the colonization of the non-SIRV strain rSC0162 and the SIRV strain rSC0163 in systemic lymphoid tissues, bacterial counts in the spleen, liver, and cecum were measured on days 3, 7, 14, and 21 post-oral immunization. The colonization levels of SIRV strain rSC0163 in the spleen, liver, and cecum on days 3 and 7 post-inoculation were comparable to those of non-SIRV strain rSC0162, indicating that the SIRV system did not affect the early colonization of *S.* Enteritidis in chickens. In contrast, on days 14 and 21 post-inoculation, colonization by SIRV strain rSC0163 in these tissues was significantly lower than that of rSC0162, because of the absence of arabinose in the chickens (Fig. 2). Specifically, SIRV strain rSC0163 was eliminated from the spleen (Fig. 2A) and liver (Fig. 2B) by days 14 and 21, whereas, it persisted in the cecum until day 21 (Fig. 2C). These results indicate that, similar to the in vitro passage results, the SIRV system mediates delayed programmed self-lysis of the *S.* Enteritidis vector in vivo under conditions of reduced arabinose availability. This colonization profile provides sufficient time for the *Salmonella* vector to effectively stimulate the lymphatic system while ensuring biosafety at later stages.

### SIRV facilitates the release of Salmonella DNA into the cytoplasm

**Fig. 3:**
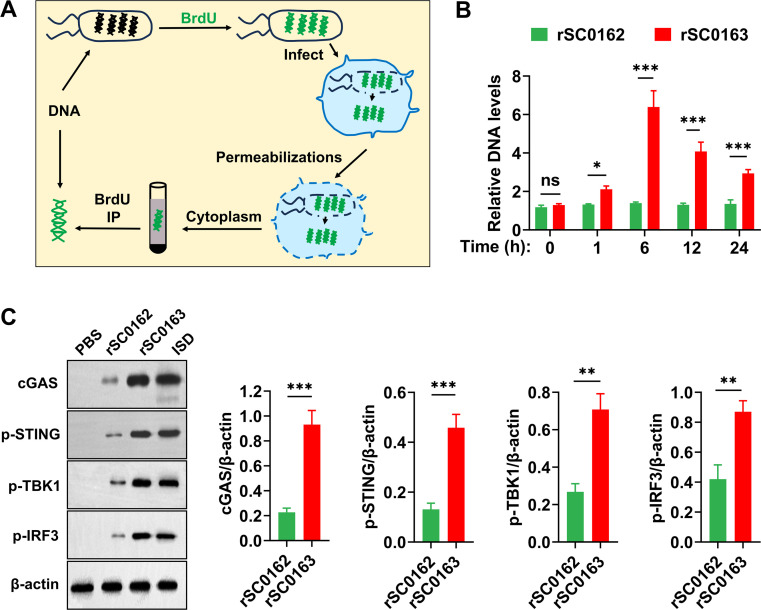
SIRV enhanced the activation of the cGAS-STING axis. RAW264.7 cells are infected with BrdU-labeled *Salmonella* strains. Cytosolic fractions are separated following infection, and BrdU-labeled bacterial DNA is retrieved using immunoprecipitation with BrdU-specific antibodies. (B) Lysates from (A) are used as templates for qRT-PCR quantification of *S.* Enteritidis DNA. (C) At 12 h post-infection with the indicated *S.* Enteritidis strains, expression of cGAS (59 kDa), p-STING (41 kDa), p-TBK1 (84 kDa), and p-IRF3 (57 kDa) in RAW264.7 cells are assessed by Western blotting. PBS is used as a negative control, ISD as a positive control, and β-actin as a loading control (42 kDa). Ns, not significant, *, *P* < 0.05, **, *P* < 0.01, ***, *P* < 0.001.

SIRV alters cell morphology and gene expression, enabling the strain to self-lyse and release cytoplasmic contents via pores formed in the cell membrane (Li et al., 2023b). To determine whether bacterium-derived DNA was released into the cytoplasm of infected cells, cytoplasmic fractions were isolated from RAW264.7 cells exposed to BrdU-labeled non-SIRV strain rSC0162 or SIRV strain rSC0163 (Fig. 3A). *Salmonella* DNA was significantly enriched in the cytoplasm of cells infected with the SIRV strain rSC0163, but not in those infected with the non-SIRV strain rSC0162, with enrichment peaking at 6 h post-infection (Fig. 3B). These findings suggested that SIRV facilitates the release of *Salmonella* DNA into the cytoplasm of host cells.

### SIRV improves innate immune responses in vivo

DNA originating from microorganisms in the cytoplasm triggers activation of the cGAS-STING axis (Li et al., 2023b). We further examined whether bacterium-derived DNA could activate the cGAS-STING axis in response to SIRV. Expression levels of cGAS, p-STING, p-TBK1, and p-IRF3 were assessed in RAW264.7 cells infected with *S.* Enteritidis strains. Infection with the SIRV strain rSC0163 resulted in increased protein expression of cGAS, p-STING, p-TBK1, and p-IRF3 compared with infection by the non-SIRV strain rSC0162. In contrast, no activation of these proteins was detected in RAW264.7 cells treated with PBS (Fig. 3C). These results indicate that the SIRV system promotes phosphorylation of STING, TBK1, and IRF3 by enhancing cGAS activity, thereby activating the cGAS-STING innate immune pathway.

### SIRV improves antibody responses to OMPs in chickens

**Fig. 4:**
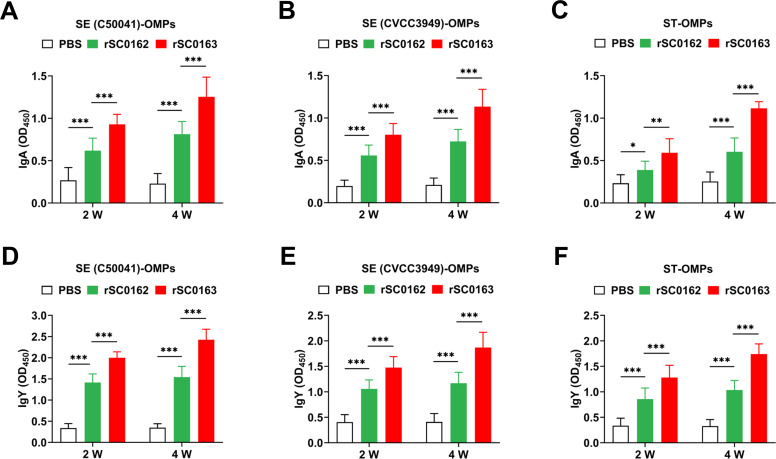
Specific mucosal and humoral immune responses induced by SIRV in chickens. (A-C) IgA antibody responses to SE (C50041)-OMPs (A), SE (CVCC3949)-OMPs (B), and ST-OMPs (C). (D-F) IgY antibody responses to SE (C50041)-OMPs (D), SE (CVCC3949)-OMPs (E), and ST-OMPs (F). Data are expressed as the mean ± SD of ten chickens. *, *P* < 0.05, **, *P* < 0.01, ***, *P* < 0.001.

To assess mucosal and humoral immune responses triggered by the SIRV system, we measured IgA and IgY antibodies in immunized chickens. Chickens orally administered the SIRV strain rSC0163 showed significantly higher levels of IgA and IgY antibodies against SE (C50041)-OMPs, SE (CVCC3949)-OMPs, and ST-OMPs at both 2 and 4 weeks post-inoculation compared with the non-SIRV strain rSC0162. Furthermore, chickens inoculated with rSC0163 exhibited higher IgA (Fig. 4A-C) and IgY (Fig. 4D-F) levels at 4 weeks than at 2 weeks post-inoculation. These results demonstrate that the SIRV system promotes both mucosal and humoral immune responses.

### SIRV improves cellular immune responses

**Fig. 5:**
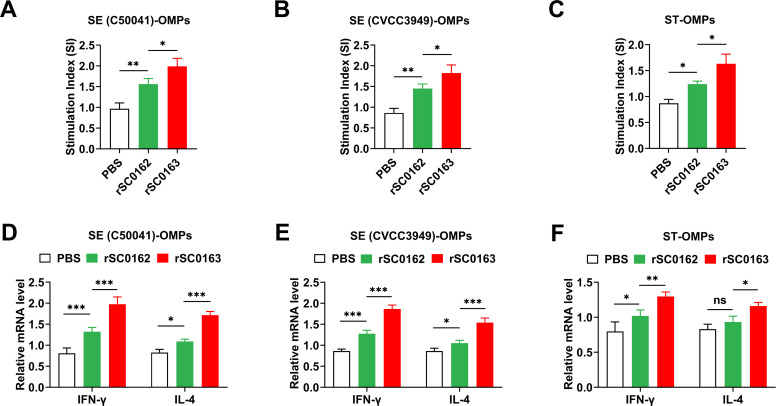
Specific cellular immune responses induced by SIRV in chickens. (A-C) Lymphocyte proliferation. PBMCs are stimulated with SE (C50041)-OMPs (A), SE (CVCC3949)-OMPs (B), and ST-OMPs (C), and proliferation is measured using the CCK-8 assay. (D-F) Cytokine production upon in vitro stimulation of lymphocytes. PBMCS are stimulated with SE (C50041)-OMPs (D), SE (CVCC3949)-OMPs (E), and ST-OMPs (F). mRNA expression levels of IFN‐γ and IL‐4 are quantified by qRT-PCR. Data are expressed as the mean ± SD of three chickens. Ns, not significant, *, *P* < 0.05, **, *P* < 0.01, ***, *P* < 0.001.

To evaluate the effect of the SIRV system on cellular immunity, we analyzed lymphocyte proliferation and cytokine production in immunized chickens. After stimulation with SE (C50041)-OMPs, SE (CVCC3949)-OMPs, and ST-OMPs, lymphocyte proliferation was significantly increased in all immunized groups, indicating the induction of cellular immune responses (Fig. 5A-C). Cytokine analysis showed that rSC0163 induced higher levels of IFN-γ and IL-4 against SE (C50041)-OMPs, SE (CVCC3949)-OMPs, and ST-OMPs compared with rSC0162 (Fig. 5D-F). IFN-γ levels were higher than IL-4 levels. These findings indicate that the SIRV system effectively enhances cellular immune responses.

### SIRV enhances the antibody-mediated bactericidal activity of phagocytes

**Fig. 6:**
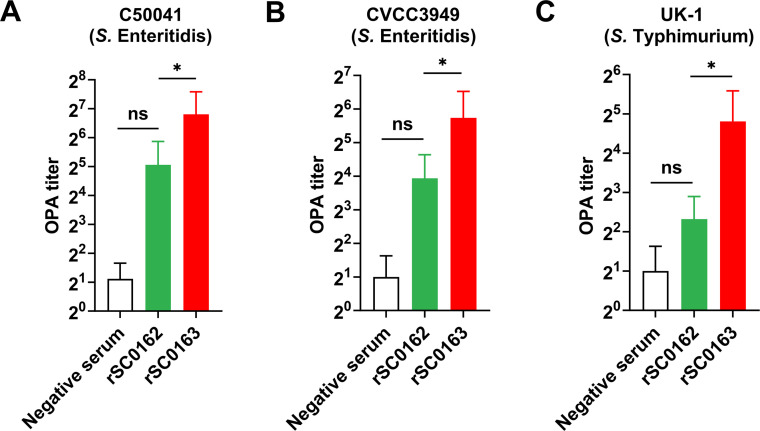
Bactericidal activity of antibodies. Opsonophagocytic activity (OPA) antibody titers against *S.* Enteritidis C50041 (A), *S.* Enteritidis CVCC3949 (B), and *S.* Typhimurium UK-1 strain (C) in chicken sera. Bacterial killing by neutrophils is assessed under opsonizing conditions. Serum from individual chickens is serially twofold diluted to determine OPA titers. Data are expressed as the mean ± SD of six chickens. Ns, not significant, *, *P* < 0.05, **, *P* < 0.01, ***, *P* < 0.001.

To evaluate whether the SIRV system improves the antibacterial function of antibodies, we assessed the opsonizing activity of chicken serum. OPA antibody titers against *S.* Enteritidis C50041 (Fig. 6A), *S.* Enteritidis CVCC3949 strain (Fig. 6B), and *S.* Typhimurium UK-1 (Fig. 6C) elicited by the SIRV strain rSC0163 were significantly higher than those induced by the non-SIRV strain rSC0162. Serum from chickens immunized with rSC0163 showed the highest OPA titers, reaching 1:256, 1:128, and 1:64, respectively. These results suggest that the SIRV system enhances the antibody-mediated opsonophagocytic killing of *Salmonella*.

### *SIRV protects chickens against S.* Enteritidis *and S.* Typhimurium *infection*

**Fig. 7:**
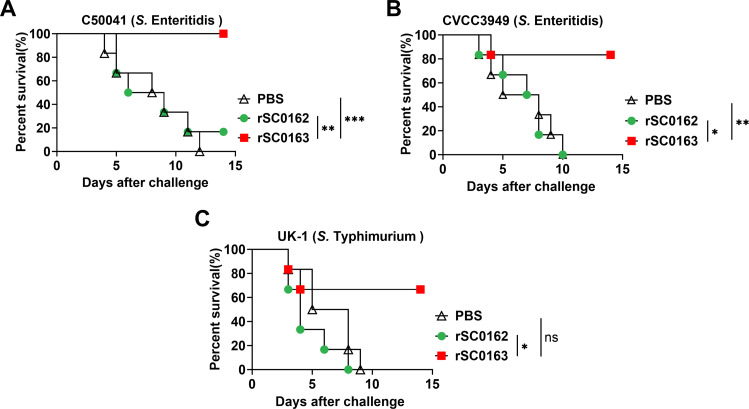
Survival rates of immunized chickens challenged with wild-type *Salmonella* strains. (A) Chickens challenged intramuscularly with *S.* Enteritidis C50041 strain. (B) Chickens challenged intramuscularly with *S.* Enteritidis CVCC3949. (C) Chickens challenged intramuscularly with *S.* Typhimurium UK-1 strain. Data are expressed as the mean ± SD of six chickens. *, *P* < 0.05, **, *P* < 0.01, ***, *P* < 0.001.

**Fig. 8:**
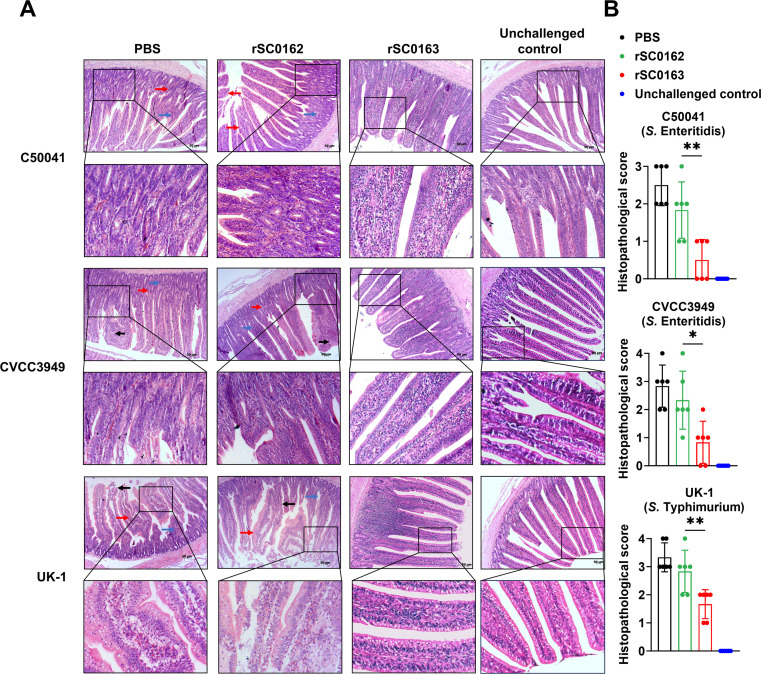
Microscopic pathological changes in the small intestine of immunized chickens infected with wild-type *Salmonella* strains. (A) Representative histopathology of small intestine tissue sections. PBS-treated and non-SIRV strain rSC0162 groups showed abnormal intestinal villi morphology (black arrows), villi hyperemia (red arrows), and inflammatory cell infiltration (blue arrows). No apparent pathological changes are observed in the SIRV strain rSC0163 group. (B) Histology scores of small intestine sections. Data are expressed as the mean ± SD. *, *P* < 0.05, **, *P* < 0.01, ***, *P* < 0.001.

To assess the cross-protection conferred by the SIRV strain rSC0163, immunized chickens were challenged with wild-type *S.* Enteritidis strains and *S.* Typhimurium strains. All chickens immunized with PBS succumbed after challenge. In contrast, rSC0163-vaccinated chickens exhibited significantly higher survival rates than those vaccinated with rSC0162 when challenged with *S.* Enteritidis C50041 (Fig. 7A), *S.* Enteritidis CVCC3949 strain (Fig. 7B), or *S.* Typhimurium UK-1 (Fig. 7C). Protection rates for rSC0163-immunized chickens were 100 %, 83 %, and 66 % against C50041, CVCC3949, and UK-1, respectively. Histological examination of small intestine tissue sections from PBS- and rSC0162- vaccinated chickens revealed abnormal villus morphology (black arrows), villus hyperemia (red arrows), and inflammatory cell infiltration (blue arrows). No significant pathological changes were observed in the rSC0163-vaccinated group (Fig. 8A-B). These results indicate that the SIRV system not only protects chickens from mortality caused by *Salmonella* challenge but also prevents inflammation associated with infection.

## Discussion

One fundamental prerequisite for the success of attenuated live vaccines is achieving a delicate balance between immunogenicity and colonization (Kong et al., 2008a). The interfering enzyme MazF from *E. coli* specifically cleaves single-stranded mRNA containing the 5′-ACA-3′ sequence (Bayles, 2014; Simanshu et al., 2013). Therefore, bacteria engineered to incorporateMazF interferase activity can undergo growth arrest, lysis, and death, thereby enhancing biosafety. Given that 98 % of the genes in the *S.* Enteritidis C50041 strain contain ACA triplets, it is probably that the SIRV system can lyse this strain (Li et al., 2023b). In the present study, rSC0163 carrying the SIRV system upregulated *mazF* expression under arabinose-reduced conditions (Fig. 1D), leading to bacterial lysis (Fig. 1E). This finding was further supported by colonization assays (Fig. 2). These results indicate that rSC0163 does not immediately self-lyse when arabinose is absent but gradually initiates self-lysis at later stages. This characteristic ensures that the SIRV strain rSC0163 has sufficient time to stimulate the lymphatic system, thereby maximizing the immune response, while also reducing its survival and transmission potential through self-lysis, significantly enhancing biosafety. Particularly in high-density farming environments, this property may lower the risk of horizontal transmission of the vaccine strain to unvaccinated poultry flocks or accidental transmission to other livestock, thereby reducing the likelihood of contamination in eggs and meat products (Kong et al., 2008b).

The activation of innate immune cells is pivotal for vaccine successand has therefore drawn significant attention in vaccine research (Coffman et al., 2010; Smedberg et al., 2014). The cGAS-STING pathway is a recently identified innate immune signaling pathway with strong immune-stimulatory capacity that promotes the maturation of APCs (Chen et al., 2022; Xu et al., 2022). In this study, we observed that cytoplasmic contents contained DNA that stimulated the cGAS-STING pathway (Fig. 3C), indicating that the SIRV system enables rSC0163 to activate this pathway. Additionally, *Salmonella*, acting as a PAMP complex, exhibits multiple innate immunostimulatory effects. For example, bacterial lipopolysaccharides and flagella enhance adaptive immune responses by activating TLR4- and TLR5-mediated pathways, respectively (Huang et al., 2021; Lu et al., 2013). Our results indicate that the SIRV strain rSC0163 enhances adjuvant efficacy by activating multiple innate immune pathways.

*Salmonella* vaccines can induce cross-immune protection against different serotypes because many common antigens (e.g., OMPs, flagellin, etc.) are highly conserved across serotypes (Alves et al., 2022; Bandholtz et al., 2002; Firmino-Cruz et al., 2022; Kang et al., 2022). In addition, live *Salmonella* vaccines can activate humoral, cellular, and mucosal immunity (Duplantis et al., 2015; Park et al., 2023). For instance, genetically engineered attenuated live *S.* Enteritidis has been shown to trigger immune responses against *S.* Typhimurium antigens, providing protection against a lethal dose of *S.* Typhimurium infection (Nandre et al., 2015). In this study, the SIRV strain rSC0163 elicited mucosal (Fig. 4A-C), humoral (Fig. 4D-F), and cellular immune responses (Fig. 5A-F) against SE (C50041)-OMPs, SE (CVCC3949)-OMPs, and ST-OMPs, and provided cross-protection against lethal doses of *Salmonella* strains (Fig. 7A-C). These results suggests that conserved antigen-mediated immune responses may be the core mechanism underlying cross-protection.

There are numerous serotypes of *Salmonella*, and cross-reactions between different serotypes or among strains within the same serotype are limited. Vaccines therefore have difficulty covering a wide range of serotypes, posing challenges for the prevention and control of this pathogen (Bamouh et al., 2024; Borowsky et al., 2009; Frost et al., 2023; Yang et al., 2019). In this study, the SIRV strain rSC0163 demonstrated a degree of cross-protection against heterologous *Salmonella* strains, whereas the non-SIRV strain rSC0162 did not. In particular, rSC0163 conferred protection against lethal doses of wild-type *S.* Enteritidis strains from different sources and the *S.* Typhimurium strain, making it a highly attractive vaccine candidate against *Salmonella*. It is worth noting that the efficacy of protective immunity can be influenced by the choice of adjuvants, which differ in their capacity to activate innate immunity (Bandholtz et al., 2002; Cribbs et al., 2003; McKee et al., 2008). For example, when chickens were challenged with a lethal dose of wild-type *S.* Enteritidis, intramuscular inoculation of rOmpF protein with QuilA adjuvant provided better protection than the same protein formulated with Al(OH)_3_ or Freund’s adjuvant (Li et al., 2020b). We reasonably speculate that rSC0163 exerts a synergistic effect on cGAS-STING pathway activation, thereby enhancing cross-reactivity. In addition, the production cost of rSC0163 is relatively low. Oral administration avoids the stress response in poultry associated with injection and minimizes growth performance decline because of stress (Embregts et al., 2016), making it highly applicable for large-scale poultry farming. Future research should aim to expand its cross-protection spectrum by evaluating the protective effects against other clinically relevant *Salmonella* serotypes, such as *Salmonella* Pullorum and *Salmonella* Gallinarum. Long-term field trials in commercial poultry farms are also required to assess vaccine durability over multiple production cycles and its performance in co-infection scenarios with common pathogens.

## Funding

This study was supported by the National Key R&D Program of China 2023YFE0123500; the Jiangsu Province Science and Technology Program Special Fund Project (BZ2022042); the Natural Science Foundation of Jiangsu Province (BK20230576); the National Natural Science Foundation of China (32302823, 32172802, 32002301, 31672516, 31172300, 30670079); the Jiangsu Excellent Postdoctoral Program (2023ZB275); the Priority Academic Program Development of Jiangsu Higher Education Institutions (PAPD) and the 111 Project (D18007).

## CRediT authorship contribution statement

**Wenjing Li:** Writing – original draft, Validation, Methodology, Investigation, Formal analysis, Data curation, Conceptualization. **Yu-an Li:** Writing – review & editing, Resources, Methodology, Funding acquisition. **Yuqin Zhang:** Investigation. **Huoying Shi:** Writing – review & editing, Supervision, Resources, Project administration, Methodology, Funding acquisition.

## Disclosures

The authors declare that they have no known competing financial interests or personal relationships that could have appeared to influence the work reported in this paper.
